# Quality of undifferentiated chest pain evaluation and diagnosis guidelines: a systematic review and critical appraisal

**DOI:** 10.1177/20542704241288955

**Published:** 2024-11-20

**Authors:** Nicole Palmer, Tejanth Pasumarthi, Joe O’Connell, Brandon Lee, Tiffany Yu, Venkata Neelima Kothapudi, Shama Patel, Rebecca L. Morgan

**Affiliations:** 1 Evidence Foundation, Cleveland Heights, OH 44106, USA; 2 Weill Cornell Medical College, New York, NY 10075, USA; 3 Faculty of Science, McMaster University, Hamilton, ON L8S 4L8, Canada; 4 Rose-Hulman Institute of Technology, Terre Haute, IN 47803, USA; 5 Department of Biological Engineering, 2167 Massachusetts Institute of Technology, Boston, MA 02139, USA; 6 Faculty of Health Sciences, McMaster University, Hamilton, ON L8S 4L8, Canada; 7 Bronxcare Health Systems, The Bronx, NY 10457, USA; 8 College of Medicine, University of Florida, Gainesville, FL 32610, USA; 9 Department of Health Research Methods, Evidence and Impact, McMaster University, Hamilton, ON L8S 4L8, Canada; 10 School of Medicine, Case Western Reserve University, Cleveland, OH 44106, USA

**Keywords:** chest pain, angina, diagnosis, guideline

## Abstract

Chest pain is a symptom that is potentially life-threatening and requires quick and accurate evaluations. This article describes the quality of guidelines related to the evaluation and diagnosis of acute, undifferentiated chest pain. After systematically evaluating existing guidelines, we found that there exists a wide variety of quality in these documents. Future documents that provide recommendations should utilize guideline evaluation tools during the creation process to ensure a high-quality product, regardless of document type.

Chest pain is the second-most common reason for presenting to the emergency department (ED) in the United States; it is the cause of more than 6.5 million and 4 million annual ED and outpatient visits, respectively.^1,2^ Chest pain is also a worldwide issue as approximately one in four individuals throughout the world will experience it in some form during their lifetime.^3^ Chest pain poses a great challenge to clinicians due to numerous etiologies that can lead to serious mortality and morbidity if left untreated.^4,5^ While more than half of all ED patients in the United States with chest pain have a non-cardiac cause, up to an approximate 15% of patients with chest pain had an underlying etiology of coronary artery disease (CAD), a condition where coronary arteries struggle to provide the heart with necessary blood flow and nutrients for optimal cardiac function.^6^ Chest pain is the most common presenting symptom of CAD.^7^ CAD affects over 18 million adults in the United States and is the leading cause of death globally, accounting for 17.9 million deaths in 2019.^8^

To account for the serious potential of a life-threatening disease in patients with undifferentiated acute chest pain, initial steps require quick and accurate evaluations. Actionable recommendations included in clinical practice guidelines can guide and standardise care. Due to the high volume of patients seen in the acute care setting and the potential for serious clinical outcomes, it is imperative that available recommendations on how to evaluate and assess the clinical presentation, as well as how to diagnose the underlying cause are rigorously developed and of high quality; however, sources of recommendations for the evaluation and diagnosis of undifferentiated chest pain vary widely, with many document types and use of inconsistent methods for the evidence review and development of recommendations. In 2011, the Institute of Medicine published Clinical Practice Guidelines We Can Trust to propose standards for creating trustworthy, transparent and standardised guidelines.^9^ However, published guidelines on undifferentiated chest pain lack clarity in underlying methods, as well as the process for determining the final recommendation.^10–12^ This study aims to understand the quality of available guidelines on undifferentiated chest pain by conducting a thorough critical appraisal.

## Materials and methods

We conducted a systematic search and critical assessment of guidelines related to the evaluation and diagnosis of acute chest pain using methods that are previously described.^13^ The protocol is registered in PROSPERO under CRD314560. This study follows guidelines for the reporting of methodology research as an adaption of the Preferred Reporting Items for Systematic Reviews and Meta-Analyses (PRISMA) guidelines for systematic reviews.^14^

We searched Ovid MEDLINE(R), Ovid EMBASE and CINAHL from January 1, 2000 through March 7, 2022 (Supplementary File S1: Search Strategy) using a strategy based on previous searches.^13^ We also searched the references of included papers and related reviews for additional documents that met the inclusion criteria.

### Eligibility

Eligible guidelines were available in English and included recommendations for the evaluation or diagnosis of patients presenting to outpatient settings with undifferentiated acute chest pain. Included guidelines did not need to refer to themselves specifically as guidelines but had applicable recommendations. Some of the included documents were iterations of previously published guidelines; all unique iterations published within the period of interest were retrieved and included.

### Screening, data extraction, and critical appraisal

Two reviewers independently and in duplicate evaluated each title and abstract of documents identified from our initial search. Two reviewers also independently evaluated each full text of the documents that fit the inclusion criteria. Any conflicts were resolved by reaching consensus between the reviewers or by consulting a third reviewer.

Specific recommendations were extracted from eligible documents by two independent reviewers along with related information (Table 1). Information used to stratify data into the reported categories, such as the type of methodology used or the document type, was extracted as well.

**Table 1.** Document characteristics summary.

Category	Number (%)	Mean (SD)
Total	30 (100%)	
Setting		
Emergency Department	15 (50%)	
Emergency care	2 (6.7%)	
Primary care	2 (6.7%)	
Unspecified	11 (36.7%)	
Document type		
Consensus paper	3 (10%)	
Guideline	23 (76.7%)	
Position Paper	2 (6.7%)	
Scientific Statement	2 (6.7%)	
WHO publishing regions		
African Region	0 (0%)	
Eastern Mediterranean Region	0 (0%)	
European Region	8 (26.7%)	
Region of the Americas	19 (63.3%)	
South-East Asian Region	1 (3.3%)	
Western Pacific Region	2 (6.7%)	
Method used		
Level of evidence	3 (10%)	
Classification of recommendations	4 (13.3%)	
Both	12 (40%)	
GRADE	2 (6.7%)	
None of the above	9 (30%)	
Applicable flow charts		1.1 (1.4)
0	13 (43.3%)	
1	8 (26.7%)	
2	4 (13.3%)	
3+	5 (16.7%)	
Population		
Paediatrics	1 (3.3%)	
Adults	1 (3.3%)	
General	28 (93.3%)	
Number of recommendations		8.9 (9.5)
Includes a systematic review	4 (13.3%)	
Collaboration between organisations	14 (46.7%)	
List of author contributions	2 (6.7%)	
Conflicts of interest statement provided	19 (63.3%)	
Patient representative used	4 (13.3%)	
Guidelines for specific populations	5 (16.7%)	

SD: standard deviation.

Three reviewers independently assessed the quality of each guideline using methods described in the Appraisal of Guidelines for Research and Evaluation II (AGREE II) tool handbook (REF). Reviewers piloted the AGREE II tool on two studies. This tool included six domains for the assessment of each guideline: ‘Scope and Purpose’, ‘Stakeholder Involvement’, ‘Rigor of Development’, ‘Clarity of Presentation’, ‘Applicability’ and ‘Editorial Independence’. A rating of the overall guideline quality was also provided. Results were then reviewed for any inconsistencies across reviewers. When inconsistencies in responses were identified, reviewers discussed the underlying evidence base for the decision to form consensus on a final judgment.

### Analysis

Scores for the AGREE II tool were calculated using the methods described in the AGREE II tool handbook. The intraclass correlation coefficient (ICC) among the three reviewers was used to calculate the inter-rater reliability of the six domains and for the overall rating of the AGREE II tool. The following subgroups were identified a priori for analysis: WHO publishing regions, method of evaluation, patient subpopulation, year of publication, and guideline author identified document type.

### Statistical analysis

Inter-rater reliability was calculated using an ICC. A Spearman's correlation coefficient (ρ) was used to measure the strength and direction of the association between the guideline's year of publication and the measure of guideline quality. In order to evaluate the effect of different categorical guideline characteristics on the measure of guideline quality, a Kruskal–Wallis test for independent samples was conducted for differences across all categories among each of the six domains and overall scoring. A Mann–Whitney *U* test was used in instances where similar groups were combined so that just two groups were compared.

### Inter-rater agreement

The average ICC across the 6 domains of the AGREE II tool was first of ‘moderate reliability’ (0.58). After correction for missed methods, the average ICC was of ‘excellent reliability’ (0.93) with the overall scoring at a ‘good reliability’ (0.76).^15^ Scoring for each category along with the ICC's 95% confidence intervals can be found in Supplementary File s2: Scaled scoring summary.

## Results

### Overview of guidelines

The initial search revealed 8611 records, which were then screened at the title and abstract stage for exclusion with 458 eligible for full-text screening. Of these full texts, 26 were found to meet all guideline requirements and were included. We present the results of the document inclusion process in a PRISMA flow diagram (Supplementary File S3: PRISMA Flow Diagram). An additional 5 guidelines were identified through a citation search of screened full-texts and related background papers. In total, 30 guidelines were included in the final analysis. The number of recommendations per guideline ranged from one to 42 with a mean of 8.9 recommendations per guideline (SD 9.5) (Table 1). Of the 30 guidelines, 11 were updates or iterations of previously published work.

Of the 30 guidelines, two focused on a specific population: one on paediatric populations and one on patients already diagnosed with Adult Congenital Heart Disease. Five additional guidelines provided unique recommendations that fit our criteria for subpopulations; all five guidelines provided relevant recommendations specific to women and two also provided recommendations for cocaine and methamphetamine users, Prinzmetal's angina and Syndrome X.

In order to evaluate if the quality of the guidelines changed over time, all guidelines were divided into 4 post hoc subgroups: 2000–2005 (*n* = 6), 2006–2011 (*n* = 5), 2012–2016 (*n* = 9), and 2017–2022 (*n* = 10). As the total range of included studies did not allow for equal stratification, one extra year was allocated to the two earlier subgroups due to the smaller number of studies in the early years when compared to the later years. A minimal meaningful change over the 22 years was defined as a change of at least five percentage points (out of the scaled score of 100) between the first and last subgroup.^13^ The included studies were also divided into subgroups based on paper type [consensus paper (*n* = 3), guideline (*n* = 23), position paper (*n* = 2), scientific statement (*n* = 2)], WHO publishing regions [European Region (*n* = 8), Region of the Americas (*n* = 19), East Asian Region (*n* = 1), and Western Pacific Region (*n* = 2)], and methods used [level of evidence (LoE) (*n* = 3), classification of recommendations (CoR) (*n* = 4), both (*n* = 12), GRADE (*n* = 2), and none of the above (*n* = 9)].

### Guideline quality

With the scaled scoring, the mean percentage values between the six domains ranged from 33.15% to 75.00% (SD 17.39). The mean of the overall scaled score was 53.15% (SD 21.43). ‘Scope and Purpose’ and ‘Clarity of Presentation’ exhibited the highest quality with 75.00% (SD 15.70) and 71.79% (SD 16.21), respectively. ‘Applicability’ and ‘Editorial Independence’ exhibited the lowest quality with 33.15% (SD 15.66) and 33.89% (SD 24.89), respectively. The remaining sections, ‘Stakeholder Involvement’ and ‘Rigor of Development’ had quality levels of 46.62% (SD 22.65) and 37.78 (SD 21.41). ‘Editorial Independence’ also had the greatest variability. Values can be found in Supplemental Figure s2: Scaled Scoring Summary.

Of the 23 AGREE II questions, the question with the highest score on average was ‘1: The overall objective(s) of the guideline is (are) specifically described’, (Mean 5.77, SD 0.93) which is found within the ‘Scope and Purpose’ domain. The question with the lowest score on average was ‘5: The views and preferences of the target population (patients, public, etc.) have been sought’, (Mean 1.91, SD 1.71), which is found within the ‘Stakeholder Involvement’ domain. Within the ‘Applicability’ domain, questions 18 and 21 were very low (Mean 2.44, SD 1.27; Mean 2.00, SD 1.30), indicating low acknowledgement of facilitators and barriers to recommendation application as well as low provision of monitoring and auditing criteria, respectively. In the ‘Editorial Independence’ domain, a low average for question 22 (Mean 2.25, SD 1.6) indicates a low frequency in reporting on the funding body and their influence on the guideline.

### Year of publication

**Figure 1.**
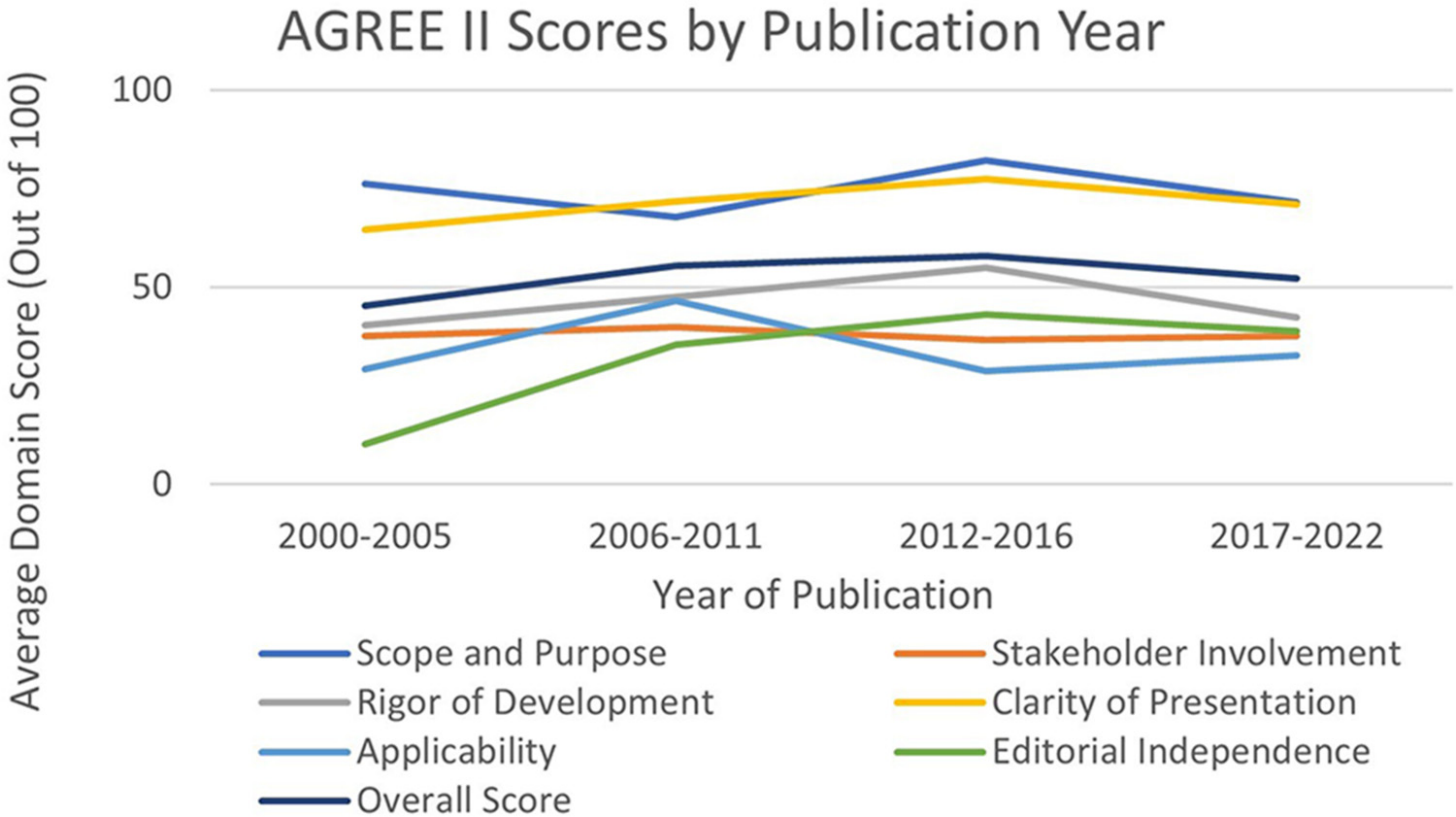
AGREE II scores by publication year.

When exploring the trends between the guideline's publication year and its quality, as assessed by AGREE II, ‘Editorial Independence’ had the largest increase in quality as time progressed, changing from 10.19% (SD 4.61) to 38.89% (SD 20.85) (Figure 1). The categories of ‘Clarity of Presentation’ and Overall Scores also increased between the first and last publication year subgroup (X% to 6.42% and X% to 6.85%, respectively).

### Region of development

Development regions were stratified based on WHO, with four subgroups represented: the Region of the Americas (*n* = 19), South-East Asian Region (*n* = 1), European Region (*n* = 8), and the Western Pacific Region (*n* = 2). Observationally, documents from the Western Pacific Region had the lowest overall average score (Mean 193.4, SD 8.35) and the article from the South-East Asian Region (415.0) had the highest average. Notable variance was seen in AGREE II values for ‘Rigor of Development’, with Western Pacific regions scoring lowest (Mean 15.62; SD 5.40) and the Americas scoring highest (Mean 51.82, SD 20.47).

### Document type

**Figure 2.**
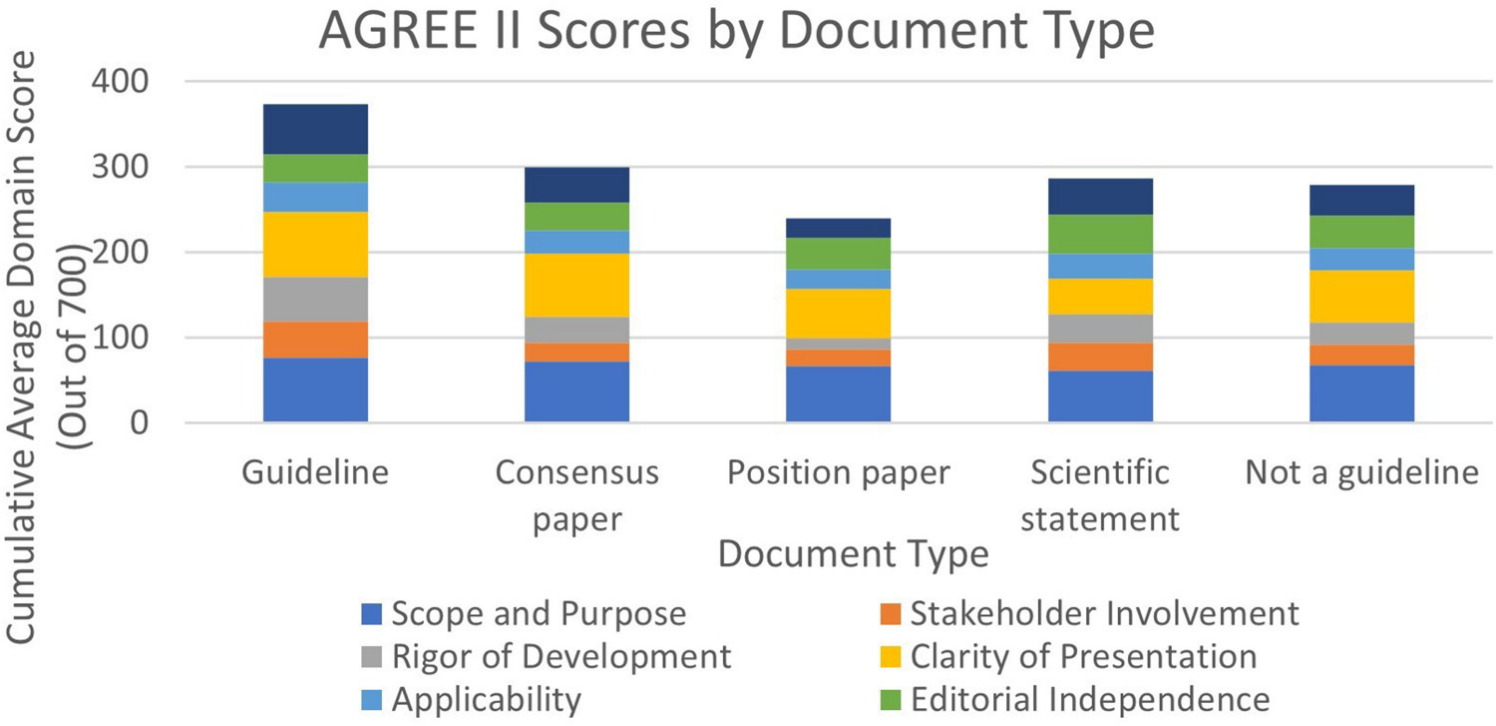
AGREE II scores by document type.

Document types are organised based on each document's own self-classification, despite all functioning as a guideline for the sake of this review. The categories are Guideline (*n* = 23), Consensus Paper (*n* = 3), Position Paper (*n* = 2) and Scientific Statement (*n* = 2). A notable difference is seen between the summed value of Guideline documents (Mean 373.50, SD 109.59) and all others, with Position Papers having the lowest summed score (Mean 239.47, SD 55.82) (Figure 2). Guideline documents had the greatest average score in all subgroups except for ‘Editorial Independence’ (Mean 32.60, SD 25.59), where Scientific Statements had the greatest value (Mean 45.83, SD 1.96).

Consensus Papers, Position Papers and Scientific Statements were combined to present documents that identified themselves as a ‘Guideline’ versus ‘Not a Guideline’, which includes the consensus papers, position papers, and scientific statement document types (*n* = 7). ‘Stakeholder Involvement’ (Guideline: Mean 41.87, SD 21.94; Not a Guideline: Mean 24.34, SD 13.24), ‘Rigor of Development’ (Guideline: Mean 52.8, SD 20.88; Not a Guideline: Mean 25.29, SD 15.87), ‘Clarity of Presentation’ (Guideline: Mean 75.28, SD 14.65; Not a Guideline: Mean 60.32, SD 16.80) and ‘Overall Scoring’ (Guideline: Mean 58.45, SD 20.16; Not a Guideline: Mean 35.71, SD 16.31) with the average Guideline having a greater score.

### Guideline development methodology

Within the 30 included guidelines, nine did not describe the use of any of these methodologies while 21 reported the use of a formal method to assess the LoR for precision and/or the evidence makeup or the CoR for benefits and treatment effect. Of these 21, four used only a CoR, three used only a LoE, 12 used both a CoR and LoE, and two used the GRADE (Grading of Recommendations Assessment, Development, and Evaluation) methodology.

**Figure 3.**
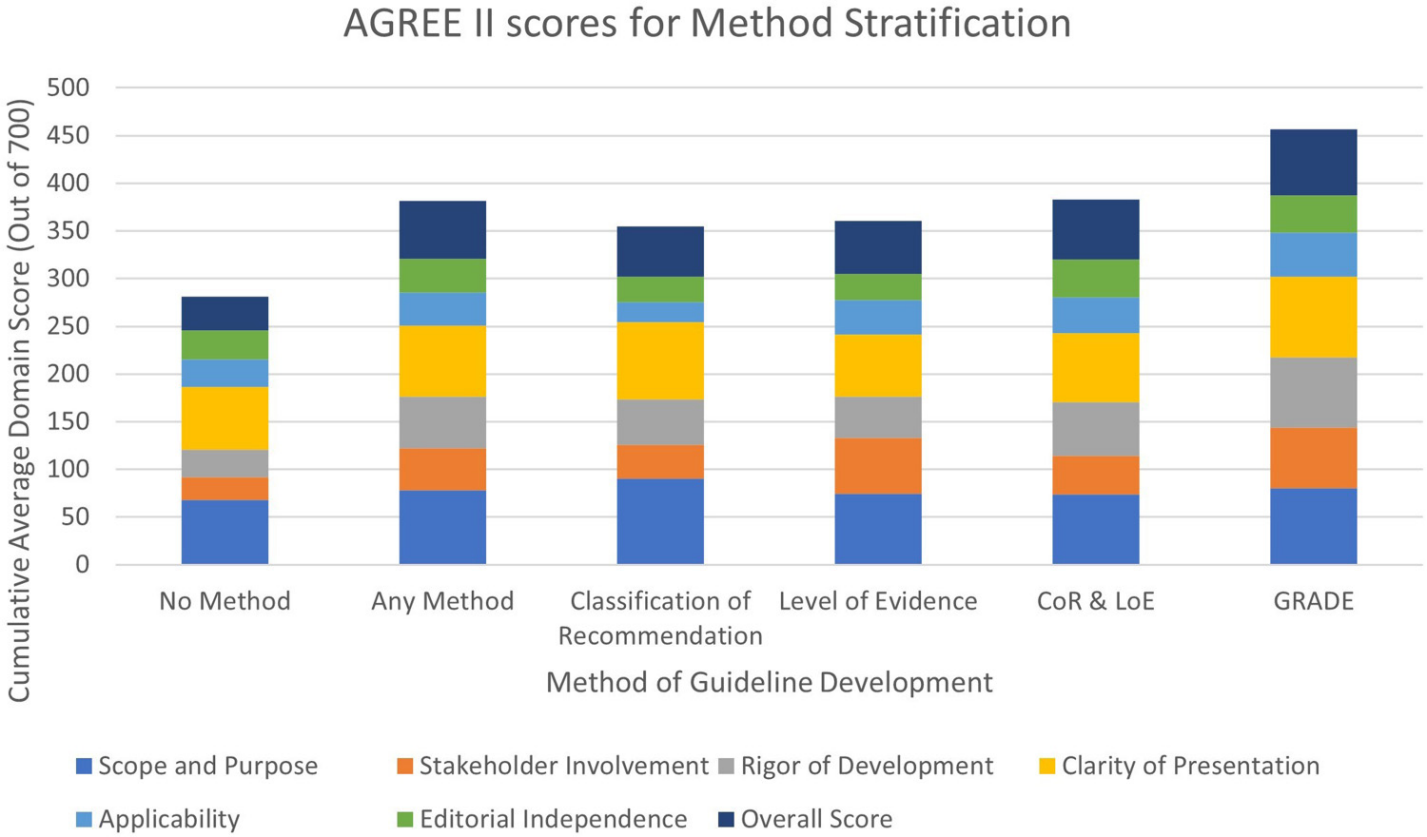
AGREE II scores by method stratification.

AGREE II scores varied most notably across guideline development methodology in four of the seven domains assessed: ‘Scope and Purpose’, ‘Stakeholder Involvement’, ‘Rigor of Development’ and Overall Scoring (Figure 3). When stratified between those that provide any formal methodology and none, scores notably across three of the same four domains: ‘Stakeholder Involvement’ (Methodology: Mean 44.00, SD 20.77; No Methodology: Mean 23.25, SD 15.63), ‘Rigor of Development’ (Methodology: Mean 54.27, SD 20.75; No Methodology: Mean 28.78, SD 16.50) and Overall Scoring (Methodology: Mean 60.58, SD 19.71; No Methodology: Mean 35.80, SD 14.46) (with greater scores for those that do have a formal methodology.

On average, guidelines that utilised GRADE had the highest score in the most categories; these included ‘Stakeholder Involvement’ (Mean 63.00, SD 34.04), ‘Rigor of Development’ (Mean 73.96, SD 25.04), ‘Clarity of Presentation’ (Mean 84.30, SD: 3.93), ‘Applicability’ (Mean 46.53, SD 32.41) and Overall Scoring (Mean 69.44, SD 35.36). For ‘Editorial Independence’, guidelines that utilised both CoR and LoE had the greatest score with a value less than 1% greater than those utilising GRADE. The methodology that had the greatest score for ‘Scope and Purpose’ was those that utilised CoE. Guidelines that did not mention the use of any formal methodology scored the lowest of all groups in ‘Scope and Purpose’ (Mean 68.31, SD 11.73), ‘Stakeholder Involvement’ (Mean 23.25, SD 15.63), ‘Rigor of Development’ (Mean 28.78, SD 16.50), ‘Clarity of Presentation’ (Mean 66.05, SD 10.48) and Overall Scoring (Mean 35.80, SD 14.46).

### Subpopulation recommendations

**Figure 4.**
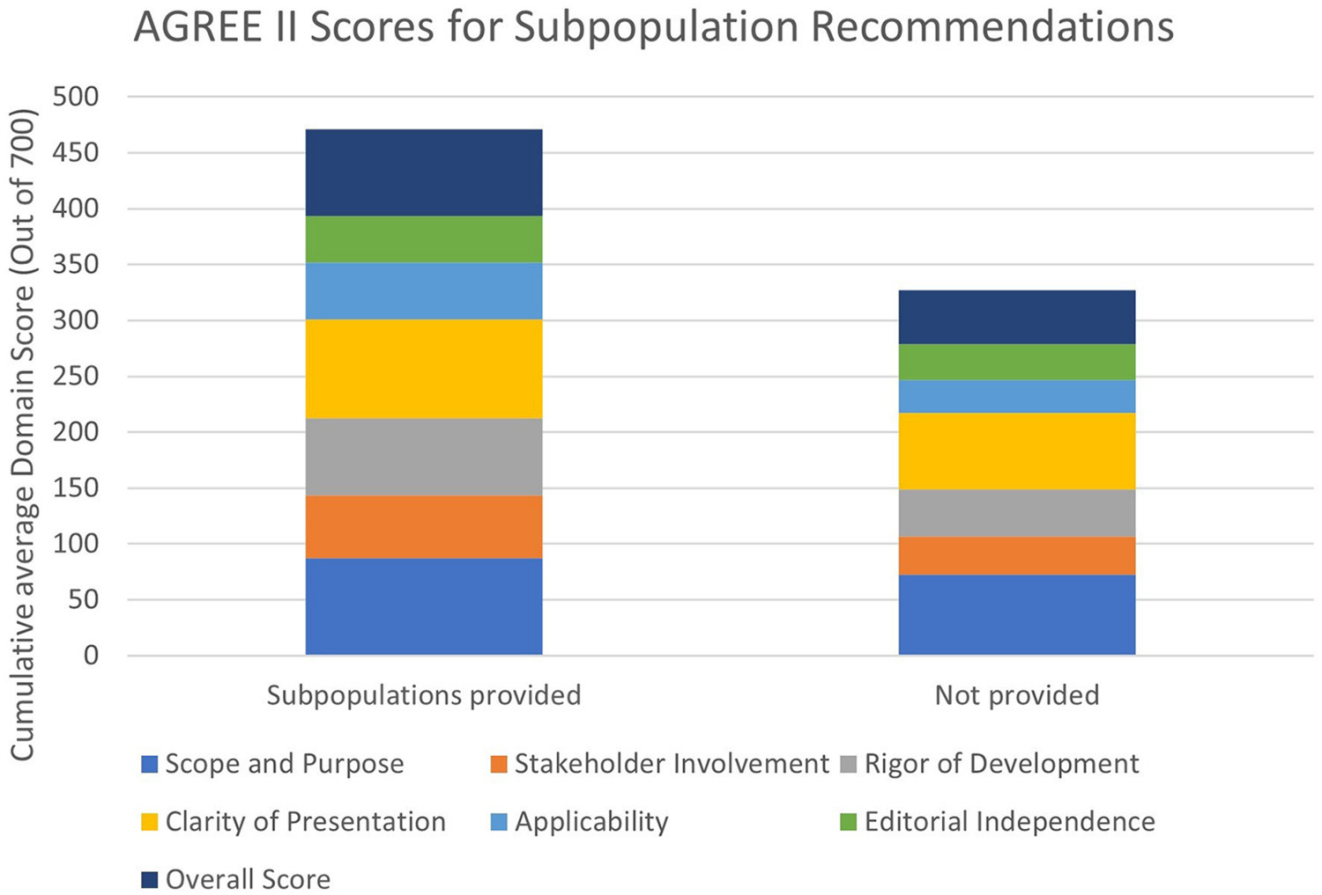
AGREE II scores for subpopulation recommendations.

Five guidelines provided specific recommendations for subpopulations of their general population that met our inclusion criteria. This included women (*n* = 5), cocaine and methamphetamine users (*n* = 2), Prinzmetal's angina (*n* = 2), and syndrome X (*n* = 2). There is a notable increase in the quality of the guidelines that provide any subpopulations in ‘Stakeholder Involvement’ (Subpopulations: Mean 56.30, SD 9.03; No Subpopulations: Mean 34.07, SD 21.32), ‘Rigor of Development’ (Subpopulations: Mean 68.75, SD 5.08; No Subpopulations: Mean 42.19, SD 22.21), ‘Clarity of Development’ (Subpopulations: Mean 88.52, SD 4.97; No Subpopulations: Mean 68.44, SD 15.60) and ‘Applicability’ (Subpopulations: Mean 50.83, SD 6.18; No Subpopulations: Mean 29.61, SD 14.55 as well as the Overall Score (Subpopulations: Mean 77.77, SD 3.93; No Subpopulations: Mean 48.22, SD 20.01) (Figure 4).

## Discussion

### Guideline quality

This study presents a critical appraisal of documents presenting recommendations for the evaluation and diagnosis of undifferentiated chest pain. There was a large variance in quality among all the guidelines evaluated and within the AGREE domains of each guideline; patterns exist related to guideline quality.

Results indicate that the guidelines only improved over the 22-year study period in ‘Editorial Independence’, which indicates a decrease in bias due to competing interests. As the domain with one of the most focused processes, a small change (i.e. mention of funding and conflicts of interest) would dramatically improve the quality of the guideline in this domain.

We hypothesised that guidelines that name themselves as a guideline as opposed to other study types would have the greatest quality level. As all these different recommendation sources are used to inform and improve the evaluation and diagnosis of patients, we believed it important to compare their quality.^16^ Indeed, guidelines have the greatest summed score of all categories and have the greatest average values for ‘Scope and Purpose’, ‘Stakeholder Involvement’, ‘Rigor of Development’, ‘Clarity of Presentation’, ‘Applicability’ and the overall assessment. A difference in AGREE II scores was also found in three categories between those that were labeled as guidelines versus those that were not, as well as the overall assessment.

Guidelines produced following the GRADE approach also had the highest quality in four of the six categories as well as the overall assessment and summed total. However, only two studies used this methodology, limiting the possible conclusions. Nevertheless, studies that used some type of formal methodology scored higher than those that did not use a methodology in three domains and had a higher quality represented in all the domains; this indicates that guidelines are of a greater quality when a formal methodology is used.

Guidelines that provided specific recommendations for relevant subpopulations scored higher in five of the seven domains. This striking difference warrants further investigation.

### Strengths and limitations

Strengths of this study include the rigorous methodology of this systematic review and the high level of reliability and validity of the AGREE II analysis. An extensive search was conducted over numerous databases and throughout the literature over the last 22 years. Evaluations at the title and abstract stage and full-text review as well as data extraction were performed in duplicate in order to decrease the impact of bias and error. To ensure that the AGREE II quality analysis was completed to a high standard and to further enhance the confidence in the results, evaluations were done in triplicate. These values were found to have excellent reliability due to the high ICC value for the six domains.

Limitations include the narrow scope of the search and the inclusion criteria. This narrow scope specific to undifferentiated chest pain required strict wording requirements. In order to distinguish undifferentiated chest pain from other similar situations, wording within recommendations was key; mentions of suspected acute coronary syndrome (ACS) did not fit our inclusion criteria but mention of symptoms that may represent/suggest ACS or are consistent with the disease model and lists chest pain or discomfort as an included symptom were included. The inclusion criteria was limited to documents in the English language for practicality and to avoid mis-translations. As the majority of guidelines were from primarily English-speaking countries, language limitations or search terms may have skewed the search to comparatively include more of these studies. Additionally, the sample size was too small to calculate significance.

### Relation to previous research

This systematic review is the first of our knowledge to systematically assess the quality of guidelines related to the evaluation and diagnosis of undifferentiated chest pain. Nevertheless, the findings of this study are in line with other guideline appraisals in overlapping fields. Our most variable domain was ‘Editorial Independence’, which was the same for the evaluation of guidelines in cardiac clinical practice.^17^ In line with past studies evaluating guidelines in both emergency medicine and emergency medicine clinical practice, ‘Scope and Purpose’ was the domain with the greatest quality and ‘Applicability’ had one of the greatest deficits.^17,18^ An evaluation of oncology guidelines and consensus statements similarly found that papers identifying as guidelines are of a greater quality than other study types providing recommendations.^19^

### Implications of findings

To our knowledge, this is the first systematic review to assess the quality of undifferentiated chest pain. Although it was found that ‘Editorial Independence’ had a modest increase in quality over time, there is still a large variability within the quality of these guidelines over the same time period. ‘Scope and Purpose’, the presentation of the guideline's aim, health questions, and target population, had a relatively consistent high score across all guidelines; ‘Applicability’, an assessment of the guideline's ability to address barriers and facilitators, resource implications, and implementation, conversely had a relatively consistent low score across all guidelines. Importantly, there were additional deficits in the number of guidelines that conducted an original systematic review to inform the recommendations (*n* = 4), those that utilised a patient representative (either a patient or designated professional) in the recommendation development process (*n* = 4) and those that provided recommendations for unique subpopulations (*n* = 5).

Consensus papers, scientific statements and position papers continue to be an important part of providing guidance to clinicians alongside guidelines, especially as they are able to address more specific situations. Nevertheless, we see a decrease in quality between guidelines and all other document types with relevant recommendations.

Key aspects of guidelines as defined by the AGREE II tool are consistently absent in the guidelines reviewed, which harms guideline end-users and resulting clinical care. Future developers of clinical guidelines addressing undifferentiated chest pain should ensure the inclusion of the following key aspects of guideline development: (1) Patient representatives; (2) Guideline updating procedure; (3) Standardised assessment of the certainty of the evidence and strength of the recommendations; (4) Original systematic reviews wherever applicable; (5) Conflict of interest disclosures; (6) Monitoring/auditing criteria and other implementation resources; and (7) Resource implications.

Subpopulation-specific recommendations, while not specifically addressed in AGREE II, may be important in topics such as undifferentiated chest pain, where different populations experience symptoms and risk levels differently than others.

Guideline development should utilise AGREE II or other guideline development tools, such as PRISMA, in order to improve the quality of clinically relevant recommendations through rigorous and standardised methods. Iterations of AGREE have been available free to the public since 2003 and should continue to be used as a valuable resource.^20^ The utility of these instruments likely would benefit other sources of recommendations that do not define themselves as guidelines and planned guidelines in the conceptual stage.

## Supplemental Material

sj-docx-1-shr-10.1177_20542704241288955 - Supplemental material for Quality of undifferentiated chest pain evaluation and diagnosis guidelines: a systematic review and critical appraisal Supplemental material, sj-docx-1-shr-10.1177_20542704241288955 for Quality of undifferentiated chest pain evaluation and diagnosis guidelines: a systematic review and critical appraisal by Nicole Palmer, Tejanth Pasumarthi, Joe O’Connell, Brandon Lee, Tiffany Yu, Venkata Neelima Kothapudi, Shama Patel and Rebecca L. Morgan in JRSM Open

sj-docx-2-shr-10.1177_20542704241288955 - Supplemental material for Quality of undifferentiated chest pain evaluation and diagnosis guidelines: a systematic review and critical appraisal Supplemental material, sj-docx-2-shr-10.1177_20542704241288955 for Quality of undifferentiated chest pain evaluation and diagnosis guidelines: a systematic review and critical appraisal by Nicole Palmer, Tejanth Pasumarthi, Joe O’Connell, Brandon Lee, Tiffany Yu, Venkata Neelima Kothapudi, Shama Patel and Rebecca L. Morgan in JRSM Open

sj-docx-3-shr-10.1177_20542704241288955 - Supplemental material for Quality of undifferentiated chest pain evaluation and diagnosis guidelines: a systematic review and critical appraisal Supplemental material, sj-docx-3-shr-10.1177_20542704241288955 for Quality of undifferentiated chest pain evaluation and diagnosis guidelines: a systematic review and critical appraisal by Nicole Palmer, Tejanth Pasumarthi, Joe O’Connell, Brandon Lee, Tiffany Yu, Venkata Neelima Kothapudi, Shama Patel and Rebecca L. Morgan in JRSM Open

sj-docx-4-shr-10.1177_20542704241288955 - Supplemental material for Quality of undifferentiated chest pain evaluation and diagnosis guidelines: a systematic review and critical appraisal Supplemental material, sj-docx-4-shr-10.1177_20542704241288955 for Quality of undifferentiated chest pain evaluation and diagnosis guidelines: a systematic review and critical appraisal by Nicole Palmer, Tejanth Pasumarthi, Joe O’Connell, Brandon Lee, Tiffany Yu, Venkata Neelima Kothapudi, Shama Patel and Rebecca L. Morgan in JRSM Open

sj-docx-5-shr-10.1177_20542704241288955 - Supplemental material for Quality of undifferentiated chest pain evaluation and diagnosis guidelines: a systematic review and critical appraisal Supplemental material, sj-docx-5-shr-10.1177_20542704241288955 for Quality of undifferentiated chest pain evaluation and diagnosis guidelines: a systematic review and critical appraisal by Nicole Palmer, Tejanth Pasumarthi, Joe O’Connell, Brandon Lee, Tiffany Yu, Venkata Neelima Kothapudi, Shama Patel and Rebecca L. Morgan in JRSM Open
