# Supplementary material for: Quality of undifferentiated chest pain evaluation and diagnosis guidelines: a systematic review and critical appraisal
Source: JRSM Open. 2024 Nov 20;15(11):20542704241288955. doi: 10.1177/20542704241288955 (PMC11772255; doi:10.1177/20542704241288955)
Supplement: sj-docx-1-shr-10.1177_20542704241288955 - Supplemental material for Quality of undifferentiated chest pain evaluation and diagnosis guidelines: a systematic review and critical appraisal [file sj-docx-1-shr-10.1177_20542704241288955.docx]

**Supplement s1** Search strategy

| Ovid MEDLINE(R) | ("Guideline"[Publication Type] OR "Guideline*"[tiab] OR "position stand"[tiab] OR "position paper"[tiab] OR "statement*"[tiab] OR "consensus"[tiab]) AND ("Chest pain*"[Mesh] OR "chest pain*"[tiab] OR "chest discomfort"[tiab] OR "angina pectoris"[Mesh] OR "Angina"[tiab] OR "Angina pectoris"[tiab] OR "stenocardia*"[tiab] OR "angor pectoris"[tiab] OR "texidor*"[tiab] OR "precordial catch"[tiab] OR "stable angina*"[tiab] OR "unstable angina*"[tiab] OR "preinfarction angina*"[tiab] OR "angina at rest"[tiab] OR "variant angina*"[tiab] OR "prinzmetal*"[tiab]) AND ("Needs assessment"[Mesh] OR "diagnosis" [Mesh] OR "symptom assessment"[Mesh] OR "APACHE"[Mesh] OR "assessment"[tiab] OR "evaluation"[tiab] OR "diagnosis"[tiab])AND (English[Language]) AND ("2000/01/01"[PDAT] : "2022/03/07"[PDAT]) |
| --- | --- |
| Ovid EMBASE | ('practice guideline' OR 'consensus' OR guideline*:ti,ab OR "position stand":ti,ab OR "position paper":ti,ab OR statement*:ti,ab OR consensus:ti,ab) AND ('angina pectoris'/exp OR 'thorax pain'/exp OR "chest pain*":ti,ab OR "chest discomfort":ti,ab OR "angina pectoris":ti,ab OR angina:ti,ab OR stenocardia*:ti,ab OR "angor pectoris":ti,ab OR "precordial catch":ti,ab OR "stable angina*":ti,ab OR "unstable angina*":ti,ab OR "preinfarction angina*":ti,ab OR "angina at rest":ti,ab OR "variant angina*":ti,ab OR prinzmetal*:ti,ab) AND ('Needs assessment'/ OR diagnosis/ OR 'symptom assessment'/ OR APACHE/exp OR assessment:ti,ab OR evaluation:ti,ab OR diagnosis:ti,ab)AND [english]/lim AND [1-1-2000]/sd NOT [3-07-2022]/sd |
| CINAHL | ((MH "consensus") OR (MH "practice guideline") OR AB guideline* OR AB "position stand" OR AB"position paper" OR AB statement* OR AB consensus) AND ((MH "Chest Pain+") OR (MH "Angina Pectoris+") OR (MH "Angina, Stable") OR (MH "Angina, Unstable") OR AB "chest pain" OR AB angina OR AB "angina pectoris" OR AB "chest discomfort" OR AB stenocardia* OR AB "angor pectoris" OR AB texidor* OR AB "precordial catch" OR AB "stable angina*" OR AB "unstable angina*" OR AB "preinfarction angina*" OR AB "angina at rest" OR AB "variant angina*" OR AB prinzmetal*) AND (MH "needs assessment" OR MH diagnosis OR MH symptom assessment OR MH APACHE+ or AB assessment OR AB evaluation OR AB diagnosis) AND LA English AND DT 20000101-20220307 |
