# Supplementary material for: Quality of undifferentiated chest pain evaluation and diagnosis guidelines: a systematic review and critical appraisal
Source: JRSM Open. 2024 Nov 20;15(11):20542704241288955. doi: 10.1177/20542704241288955 (PMC11772255; doi:10.1177/20542704241288955)
Supplement: sj-docx-2-shr-10.1177_20542704241288955 - Supplemental material for Quality of undifferentiated chest pain evaluation and diagnosis guidelines: a systematic review and critical appraisal [file sj-docx-2-shr-10.1177_20542704241288955.docx]

**Supplement s2** Scaled scoring summary

| **Author** | **Year** | **Scope and Purpose** | **Stakeholder Involvement** | **Rigor of Development** | **Clarity of Presentation** | **Applicability** | **Editorial Independence** | **Overall Rating** |
| --- | --- | --- | --- | --- | --- | --- | --- | --- |
| Albus | 2017 | 79.63 | 61.11 | 44.44 | 72.22 | 33.33 | 8.33 | 66.67 |
| Amsterdam | 2010 | 61.11 | 44.44 | 47.22 | 50.00 | 44.44 | 44.44 | 50.00 |
| Anderson | 2012 | 77.78 | 53.70 | 75.69 | 90.74 | 52.78 | 80.56 | 77.78 |
| Anderson | 2011 | 79.63 | 50.00 | 71.53 | 90.74 | 58.33 | 52.78 | 72.22 |
| Arntz | 2005 | 72.22 | 37.04 | 22.92 | 53.7 | 33.33 | 5.56 | 27.78 |
| Aroney | 2001 | 48.15 | 35.19 | 19.44 | 38.89 | 18.06 | 5.56 | 16.67 |
| Beache | 2020 | 79.63 | 38.89 | 56.25 | 81.48 | 23.61 | 11.11 | 44.44 |
| Braunwald | 2000 | 92.59 | 53.7 | 63.89 | 83.33 | 44.44 | 16.67 | 77.78 |
| Braunwald | 2002 | 92.59 | 51.85 | 63.89 | 83.33 | 44.44 | 16.67 | 77.78 |
| Budoff | 2017 | 62.96 | 20.37 | 20.14 | 33.33 | 13.89 | 47.22 | 33.33 |
| Campbell | 2014 | 83.33 | 57.41 | 75.69 | 90.74 | 43.06 | 55.56 | 66.67 |
| Casagranda | 2013 | 74.07 | 14.81 | 35.42 | 68.52 | 27.78 | 38.89 | 38.89 |
| Cesar | 2014 | 62.96 | 46.30 | 41.67 | 50.00 | 25.00 | 61.11 | 50.00 |
| Chessa | 2021 | 83.33 | 40.74 | 36.81 | 77.78 | 29.17 | 50.00 | 50.00 |
| Cooper | 2010 | 96.30 | 77.78 | 66.67 | 85.19 | 55.56 | 69.44 | 83.33 |
| Crocco | 2002 | 70.37 | 24.07 | 6.94 | 55.56 | 20.83 | 5.56 | 16.67 |
| Erhardt | 2002 | 72.22 | 18.52 | 40.97 | 55.56 | 30.56 | 5.56 | 44.44 |
| Fesmire | 2000 | 81.48 | 42.59 | 47.92 | 70.37 | 16.67 | 11.11 | 38.89 |
| Fihn | 2012 | 87.04 | 42.59 | 61.81 | 90.74 | 50.00 | 47.22 | 72.22 |
| Fox | 2006 | 53.70 | 25.93 | 40.28 | 66.67 | 29.17 | 8.33 | 44.44 |
| Gulati | 2021 | 94.44 | 72.22 | 68.75 | 94.44 | 54.17 | 41.67 | 83.33 |
| Hoffman | 2015 | 94.44 | 16.67 | 40.97 | 81.48 | 9.72 | 2.78 | 44.44 |
| Hoffman | 2012 | 94.44 | 16.67 | 40.97 | 81.48 | 9.72 | 2.78 | 44.44 |
| Kim | 2015 | 75.93 | 31.48 | 88.19 | 72.22 | 22.22 | 52.78 | 72.22 |
| Liew | 2011 | 48.15 | 1.85 | 11.81 | 66.67 | 45.83 | 2.78 | 27.78 |
| Musey | 2021 | 81.48 | 87.04 | 91.67 | 87.04 | 69.44 | 66.67 | 94.44 |
| Pontone | 2022 | 57.41 | 11.11 | 43.06 | 83.33 | 29.17 | 44.44 | 55.56 |
| Porter | 2018 | 37.04 | 16.67 | 33.33 | 57.41 | 27.78 | 44.44 | 50.00 |
| Rybicki | 2015 | 88.89 | 50.00 | 33.33 | 72.22 | 19.44 | 47.22 | 55.56 |
| Stepinska | 2020 | 62.96 | 16.67 | 17.36 | 61.11 | 23.61 | 69.44 | 27.78 |
| Zuin | 2017 | 75.93 | 12.96 | 12.50 | 61.11 | 22.22 | 5.56 | 16.67 |
| Mean (SD) | N/A | 75.00 (15.70) | 37.78 (21.41) | 46.62 (22.65) | 71.79 (16.21) | 33.15 (15.66) | 33.89 (24.89) | 53.15 (21.43) |
| ICC (95% CI) | N/A | 0.87 (0.78, 0.93) | 0.94 (0.90, 0.97) | 0.99 (0.99, 1.00) | 0.92 (0.85, 0.95) | 0.93 (0.87, 0.96) | 0.93 (0.88, 0.96) | 0.76 (0.62, 0.87) |

SD: standard deviation

ICC: intra-correlation coefficient

CI: confidence interval
