# Supplementary figures and images for: Quality of undifferentiated chest pain evaluation and diagnosis guidelines: a systematic review and critical appraisal
Source: JRSM Open. 2024 Nov 20;15(11):20542704241288955. doi: 10.1177/20542704241288955 (PMC11772255; doi:10.1177/20542704241288955)

**Supplement s3** PRISMA flow diagram


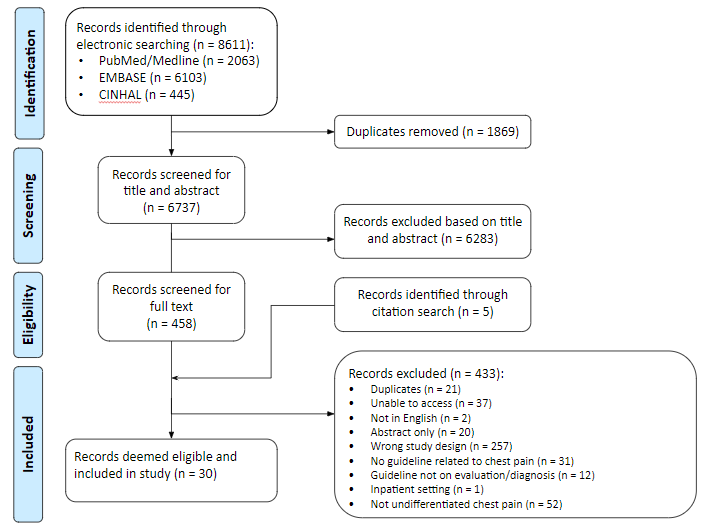

Supplement: sj-docx-3-shr-10.1177_20542704241288955 - Supplemental material for Quality of undifferentiated chest pain evaluation and diagnosis guidelines: a systematic review and critical appraisal [file sj-docx-3-shr-10.1177_20542704241288955.docx]
