# Supplementary material for: Quality of undifferentiated chest pain evaluation and diagnosis guidelines: a systematic review and critical appraisal
Source: JRSM Open. 2024 Nov 20;15(11):20542704241288955. doi: 10.1177/20542704241288955 (PMC11772255; doi:10.1177/20542704241288955)
Supplement: sj-docx-4-shr-10.1177_20542704241288955 - Supplemental material for Quality of undifferentiated chest pain evaluation and diagnosis guidelines: a systematic review and critical appraisal [file sj-docx-4-shr-10.1177_20542704241288955.docx]

**Supplement s4** Study specifics

| **Author** | **Title** | **Country** | **Developing institution** | **Type of document** | **Population** | **Setting of patient presentation** | **Funding source** |
| --- | --- | --- | --- | --- | --- | --- | --- |
| Albus 2017 | The Diagnosis of Chronic Coronary Heart Disease | Germany | German National Disease Management Chronic Heart Disease development group | Guideline | General | Primary care | N/A |
| Amsterdam 2010 | Testing of low-risk patients presenting to the emergency department with chest pain: a scientific statement from the American Heart Association | USA | American Heart Association | Scientific Statement | General | Emergency care | N/A |
| Anderson 2012 | 2012 ACCF/AHA focused update incorporated into the ACCF/AHA 2007 guidelines for the management of patients with unstable angina/non-ST-elevation myocardial infarction: a report of the American College of Cardiology Foundation/American Heart Association Task Force on Practice Guidelines | USA | American College of Cardiology Foundation/American Heart Association | Guideline | General | Unknown | N/A |
| Anderson 2011 | 2011 ACCF/AHA Focused Update Incorporated Into the ACC/AHA 2007 Guidelines for the Management of Patients With Unstable Angina/Non-ST-Elevation Myocardial Infarction: a report of the American College of Cardiology Foundation/American Heart Association Task Force on Practice Guidelines | USA | American College of Cardiology Foundation/American Heart Association/American College of Emergency Physicians, Society for Cardiovascular Angiography and Interventions, and Society of Thoracic Surgeons | Guideline | General | Unknown | N/A |
| Aroney 2001 | Current guidelines for the management of unstable angina: a new diagnostic and management paradigm | Australia | National Heart Foundation Australia, Cardiac Society of Australia and New Zealand | Guideline | General | Unknown | N/A |
| Beache 2020 | ACR Appropriateness Criteria® Acute Nonspecific Chest Pain-Low Probability of Coronary Artery Disease | USA | American College of Radiology | Guideline | General | Unknown | N/A |
| Braunwald 2002 | ACC/AHA 2002 guideline update for the management of patients with unstable angina and non-ST-segment elevation myocardial infarction--summary article: a report of the American College of Cardiology/American Heart Association task force on practice guideline | USA | American College of Cardiology Foundation/American Heart Association | Guideline | General | Unknown | N/A |
| Braunwald 2000 | ACC/AHA guidelines for the management of patients with unstable angina and non-ST-segment elevation myocardial infarction. A report of the American College of Cardiology/American Heart Association Task Force on Practice Guidelines (Committee on the Management of Patients With Unstable Angina) | USA | American College of Cardiology Foundation/American Heart Association | Guideline | General | Unknown | N/A |
| Budoff 2017 | Assessment of Coronary Artery Disease by Cardiac Computed Tomography | USA | American Heart Association | Scientific Statement | General | Unknown | N/A |
| Campbell 2014 | ACC/AAP/AHA/ASE/HRS/ SCAI/SCCT/SCMR/SOPE 2014 Appropriate Use Criteria for Initial Transthoracic Echocardiography in Outpatient Pediatric Cardiology | USA | American College of Cardiology Appropriate Use Criteria Task Force, American Academy of Pediatrics, American Heart Association, American Society of Echocardiography, Heart Rhythm Society, Society for Cardiovascular Angiography and Interventions, Society of Cardiovascular Computed Tomography, Society for Cardiovascular Magnetic Resonance, and Society of Pediatric Echocardiography | Guideline | Pediatrics | Emergency department | N/A |
| Casagranda 2013 | Proposal for the use in emergency departments of cardiac troponins measured with the latest generation methods in patients with suspected acute coronary syndrome without persistent ST-segment elevation | Italy | N/A | Guideline | General | Emergency department | N/A |
| Cesar 2014 | Guideline for Stable Coronary Artery Disease | Brazil | Sociedade Brasileira de Cardiologia | Guideline | General | Unknown | N/A |
| Chessa 2021 | Emergency department management of patients with adult congenital heart disease: a consensus paper from the ESC Working Group on Adult Congenital Heart Disease, the European Society for Emergency Medicine (EUSEM), the European Association for Cardio-Thoracic Surgery (EACTS), and the Association for Acute Cardiovascular Care (ACVC) | Italy | European Society of Cardiology, European Society for Emergency Medicine, European Association for Cardio-Thoracic Surgery, and the Association for Acute Cardiovascular Care | Consensus paper | Patients with Adult Congenital Heart Disease | Emergency department | N/A |
| Cooper 2010 | Chest pain of recent onset: Assessment and diagnosis of recent onset chest pain or discomfort of suspected cardiac origin | UK | National Institute for Health | Guideline | General | Unknown | N/A |
| Crocco 2002 | Prehospital triage of chest pain patients | USA | National Association of EMS Physicians | Position paper | General | Emergency care | N/A |
| Erhardt 2002 | Task force on the management of chest pain | Sweden | European Society of Cardiology | Guideline | General | Emergency department | The European Society of Cardiology |
| Fesmire 2000 | Clinical Policy: Critical Issues in the Evaluation and Management of Adult Patients Presenting With Suspected Acute Myocardial Infarction or Unstable Angina | USA | American College of Emergency Physicians, Emergency Medicine Residents' Association, Emergency Nurses Association | Guideline | General | Emergency department | N/A |
| Fihn 2012 | 2012 ACCF/AHA/ACP/AATS/PCNA/SCAI/STS Guideline for the diagnosis and management of patients with stable ischemic heart disease: a report of the American College of Cardiology Foundation/American Heart Association Task Force on Practice Guidelines, and the | USA | American College of Cardiology Foundation, American Heart Association, American College of Physicians,  American Association for Thoracic Surgery,  Preventive Cardiovascular Nurses Association, The Society for Cardiovascular Angiography and Interventions, Society of Thoracic Surgeons | Guideline | General | Emergency care | N/A |
| Fox 2006 | Guidelines on the management of stable angina pectoris: executive summary | UK | European Society of Cardiology | Guideline | General | Unknown | NA |
| Gulati 2021 | 2021 AHA/ACC/ASE/CHEST/SAEM/SCCT/SCMR Guideline for the Evaluation and Diagnosis of Chest Pain: A Report of the American College of Cardiology/American Heart Association Joint Committee on Clinical Practice Guidelines | USA | American Heart Association, American College of Cardiology, American Society of Echocardiography, American College of Chest Physicians, Society for Academic Emergency Medicine, Society of Cardiovascular Computed Tomography, Society for Cardiovascular Magnetic Resonance | Guideline | General | Emergency department | N/A |
| Hoffman 2015 | ACR Appropriateness Criteria Acute Nonspecific Chest Pain-Low Probability of Coronary Artery Disease. | USA | American College of Radiology | Guideline | General | Emergency department | N/A |
| Hoffman 2012 | ACR Appropriateness Criteria((R)) Acute Nonspecific Chest Pain-Low Probability of Coronary Artery Disease | USA | American College of Radiology | Guideline | General | Emergency department | N/A |
| Kim 2015 | Korean guidelines for the appropriate use of cardiac CT. | Korea | Korean Society of Radiology, Korean Society of Cardiology | Guideline | General | Emergency department | National Strategic Coordinating Center for Clinical Research |
| Liew 2011 | Noninvasive Coronary Artery Imaging: Current Clinical Applications. Cardiac Society of Australia and New Zealand Guidelines | Australia and New Zealand | The Cardiac Society of Australia and New Zealand | Guideline | General | Emergency department | N/A |
| Musey Jr 2021 | Guidelines for reasonable and appropriate care in the  emergency department (GRACE): Recurrent, low-risk chest  pain in the emergency department | USA | Society for Academic Emergency Medicine | Guideline | General | Emergency department | Society for Academic Emergency Medicine |
| Pontone 2022 | Clinical applications of cardiac computed  tomography: a consensus paper of the European  Association of Cardiovascular Imaging—part I | International | European Association of Cardiovascular Imaging | Consensus paper | General | Unknown | N/A |
| Porter 2018 | Clinical Applications of Ultrasonic Enhancing Agents in Echocardiography: 2018 American Society of Echocardiography Guidelines Update | USA | American Society of Echocardiography | Guideline | General | Emergency department | N/A |
| Rybicki 2015 | 2015 ACR/ACC/AHA/AATS/ACEP/ ASNC/NASCI/SAEM/SCCT/SCMR/ SCPC/SNMMI/STR/STS Appropriate Utilization of Cardiovascular Imaging in Emergency Department Patients With Chest Pain | USA | American College of Radiology, American College of Cardiology, American Heart Association, American Association  for Thoracic Surgery, American College of Emergency Physicians, American Society of Nuclear Cardiology, North American Society for Cardiovascular Imaging, Society for Academic Emergency Medicine, Society of Cardiovascular Computed Tomography, Society for Cardiovascular Magnetic Resonance, Society of Cardiovascular Patient Care, Society of Nuclear Medicine and Molecular Imaging,  Society of Thoracic Radiology, Society of Thoracic Surgeons | Guideline | General | Emergency department | N/A |
| Stepinska 2020 | Diagnosis and risk stratification of chest pain patients in the emergency department: focus on acute coronary syndromes. A position paper of the Acute Cardiovascular Care Association | Poland | Acute Cardiovascular Care Association | Position paper | General | Emergency department | N/A |
| Zuin 2017 | ANMCO-SIMEU Consensus Document: in-hospital  management of patients presenting with chest pain | International | Associazione Nazionale Medici Cardiologi Ospedalieri, Società Italiana Medicina d'Emergenza, European Society of Cardiology | Consensus document | General | Emergency department | N/A |
