# Supplementary material for: Quality of undifferentiated chest pain evaluation and diagnosis guidelines: a systematic review and critical appraisal
Source: JRSM Open. 2024 Nov 20;15(11):20542704241288955. doi: 10.1177/20542704241288955 (PMC11772255; doi:10.1177/20542704241288955)
Supplement: sj-docx-5-shr-10.1177_20542704241288955 - Supplemental material for Quality of undifferentiated chest pain evaluation and diagnosis guidelines: a systematic review and critical appraisal [file sj-docx-5-shr-10.1177_20542704241288955.docx]

**Supplement s5** Included guideline citations

| - Albus C, Barkhausen J, Fleck E, et al. The Diagnosis of Chronic Coronary Heart Disease. *Dtsch Arztebl Int* 2017; 114: 712–9. - Amsterdam E, Kirk JD, Bluemke DA, et al. Testing of low-risk patients presenting to the emergency department with chest pain: a scientific statement from the American Heart Association. *Circulation* 2010; 122: 1756-1776. - Anderson JL, Adams CD, Antman EM, et al. 2012 ACCF/AHA Focused Update Incorporated Into the ACCF/AHA 2007 Guidelines for the Management of Patients With Unstable Angina/Non–ST-Elevation Myocardial Infarction. *Circulation* 2013; 127: e663-828. - Anderson JL, Adams CD, Antman EM, et al. 2011 ACCF/AHA Focused Update Incorporated Into the ACC/AHA 2007 Guidelines for the Management of Patients With Unstable Angina/Non-ST-Elevation Myocardial Infarction. *Circulation* 2011; 123: e426-579. - Aroney C, Boyden AN, Jelinek MV, et al. Current guidelines for the management of unstable angina: a new diagnostic and management paradigm. *Intern Med J* 2001; 31: 104-11. - Beache GM, Mohammed TH, Hurwitz Koweek LM, et al. ACR Appropriateness Criteria® Acute Nonspecific Chest Pain-Low Probability of Coronary Artery Disease. *J Am Coll Radiol.* 2020; 17:S346-S354. - Braunwald, Antman EM, Beasley JW, et al. ACC/AHA guidelines for the management of patients with unstable angina and non-ST-segment elevation myocardial infarction. *J Am Coll Cardiol.* 2000; 36:970-1062. - Braunwald E, Antman EM, Beasley JW, et al. ACC/AHA 2002 guideline update for the management of patients with unstable angina and non-ST-segment elevation myocardial infarction--summary article. *J Am Coll Cardiol.* 2002; 40:1366-74. - Budoff JM, Achenbach S, Blumenthal SR, et al. Assessment of Coronary Artery Disease by Cardiac Computed Tomography. *Circulation.* 2017; 114:1761-1791. - Campbell RM, Douglas PS, Eidem BW, et al. ACC/AAP/AHA/ASE/HRS/SCAI/SCCT/SCMR/SOPE 2014 appropriate use criteria for initial transthoracic echocardiography in outpatient pediatric cardiology. *J Am Soc Echocardiogr*. 2014; 27:1247-1266. - Casagranda I, Cavazza M, Clerico A, et al. Proposal for the use in emergency departments of cardiac troponins measured with the latest generation methods in patients with suspected acute coronary syndrome without persistent ST-segment elevation. *Clin Chem Lab Med*. 2013; 1727-37. - Cesar LA, Ferreira JF, Armaganijan D, et al. Guideline for stable coronary artery disease. *Arq Bras Cardiol.* 2014; 103:1-56. - Chessa M, Brida M, Gatzoulis MA, et al. Emergency department management of patients with adult congenital heart disease. *Eur.* 2021; 42:2527-2535. - Cooper A, Calvert N, Skinner J, et al., Chest Pain of Recent Onset. Report, National Clinical Guideline Centre for Acute and Chronic Conditions, UK, Mar 2010. - Crocco TJ, Sayre MR, Aufderheide TP. Prehospital triage of chest pain patients. *Prehosp Emerg Care.* 2002; 6:224-8. - Erhardt L, Herlitz J, Bossaert L, et al. Task force on the management of chest pain. *Eur Heart J*. 2002; 23:1153-76. - Fesmire FM, Campbell M, Decker WW, et al. Clinical Policy: Critical Issues in the Evaluation and Management of Adult Patients Presenting With Suspected Acute Myocardial Infarction or Unstable Angina. *Ann Emerg Med*. 2000; 35:521-544. - Fihn SD, Gardin JM, Abrams J, et al. 2012 ACCF/AHA/ACP/AATS/PCNA/SCAI/STS Guideline for the diagnosis and management of patients with stable ischemic heart disease. *J Am Coll Cardiol.* 2012; 60:e44-e164. - Fox K, Alonso-Garcia MA, Ardissino D, et al. Guidelines on the management of stable angina pectoris: executive summary. *Eur.* 2006; 27:1341-1381. - Gulati M, Levy PD, Mukherjee D, et al. 2021 AHA/ACC/ASE/CHEST/SAEM/SCCT/SCMR Guideline for the Evaluation and Diagnosis of Chest Pain. *J Am Coll Cardiol.* 2021; 78:e187-e285. - Hoffmann U, Akers SR, Brown RK, et al. ACR Appropriateness Criteria Acute Nonspecific Chest Pain-Low Probability of Coronary Artery Disease. *J Am Coll Radiol.* 2015; 12:1266-71. - Hoffmann U, Venkatesh V, White RD, et al. ACR Appropriateness Criteria((R)) Acute Nonspecific Chest Pain-Low Probability of Coronary Artery Disease. *J Am Coll Radiol.* 2012; 9:745-750. - Kim YJ, Yong HS, Kim SM, et al. Korean guidelines for the appropriate use of cardiac CT. *Korean J Radiol.* 2015; 16:251-85. - Liew GYH, Feneley M, Worthley SG. Noninvasive Coronary Artery Imaging: Current Clinical Applications. *Heart Lung Circ*. 2011; 20:425-437. - Musey PI Jr, Bellolio F, Upadhye S, et al. Guidelines for reasonable and appropriate care in the emergency department (GRACE): Recurrent, low-risk chest pain in the emergency department. *Acad Emerg Med*. 2021; 28:718-744. - Pontone G, Rossi A, Guglielmo M, et al. Clinical applications of cardiac computed tomography: a consensus paper of the European Association of Cardiovascular Imaging-part I. *Eur Heart J Cardiovasc Imaging*. 2022; 23:299-314. - Porter TR, Mulvagh SL, Abdelmoneim SS, et al. Clinical Applications of Ultrasonic Enhancing Agents in Echocardiography: 2018 American Society of Echocardiography Guidelines Update. *J Am Soc Echocardiogr.* 2018; 31:P241-274. - Rybicki FJ, Udelson JE, Peacock WF, et al. 2015 ACR/ACC/AHA/AATS/ACEP/ ASNC/NASCI/SAEM/SCCT/SCMR/ SCPC/SNMMI/STR/STS Appropriate Utilization of Cardiovascular Imaging in Emergency Department Patients With Chest Pain. *J Am Coll Cardiol*. 2015; 67:853-79. - Stepinska J, Lettino M, Ahrens I, et al. Diagnosis and risk stratification of chest pain patients in the emergency department. *Eur Heart J Acute Cardiovasc.* 2020; 9:76-89. - Zuin G, Parato VM, Groff P, et al. ANMCO/SIMEU Consensus Document: In-hospital management of patients presenting with chest pain. *Giornale Italiano di Cardiologia*. 2016; 17:416-446. |
| --- |
